# Sensory neural hearing loss in Behcet's disease successfully controlled with infliximab, case report and review of literature

**DOI:** 10.1002/ccr3.6457

**Published:** 2022-10-12

**Authors:** Mohanad Faisal, Haidar Barjas, Wisam Alwassiti, Walid Omer, Nabeel Abdulla

**Affiliations:** ^1^ Hamad Medical Corporation Doha Qatar

**Keywords:** audiology, Behcet's disease, hearing loss, rheumatology

## Abstract

Behcet's disease is a systemic autoimmune disorder occasionally associated with otological manifestations, including sensorineural hearing loss. We are reporting a case of Behcet's disease, which was complicated by sensorineural hearing loss and managed successfully with anti‐TNF agent Infliximab.

## Graphical abstract

To provide review about the interesting association between Bechet's disease and sensory‐neural hearing loss and the possibility of managing this manifestation of Behcet's disease with anti‐TNF agent like infliximab.

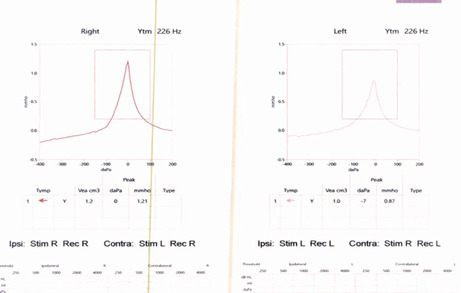

## 1 INTRODUCTION

Behcet's disease is a vasculitis affecting small to medium and even large vessels (arteries and veins). It is primarily characterized by recurrent oral ulcers, genital ulcers, skin and ophthalmological manifestations such as uveitis. It is most prevalent in the ancient silk road from Eastern Asia to the Mediterranean. It is common in Turkey, with a prevalence of 350/100,000 population. Cases have been reported in Northern America and Europe as well. This syndrome can affect males and females in most regions, typically between 20 and 40 years of age at the onset of the disease. Carriers of HLA‐B5 and HLA‐B51 are more prone to develop Behcet's disease than non‐carriers. It is thought that an infectious or environmental antigen triggering an autoimmune response in genetically predisposed individuals is behind its pathogenesis. Diagnostic criteria for Behcet's disease require the presence of oral ulcers and any of the following: genital ulcers, typical eye lesions, typical skin lesions, or a positive pathergy test.^1^ Treatment of Behcet's disease depends on its manifestation, whether oro‐genital ulcers, ocular disease, vasculitis, arthritis, gastrointestinal or neurological manifestations. Recurrent oro‐genital ulcers are usually treated with colchicine alone. At the same time, significant manifestations like posterior uveitis, vasculitis, and gastrointestinal disease usually require high‐dose glucocorticoid along with another immunosuppressive agent.^2^

Here, we present a 37‐year‐old Middle Eastern male patient with Behcet's disease with recurrent episodes of bilateral sensorineural hearing loss that responded well to oral prednisolone, Infliximab, and Azathioprine.

## 2 CASE PRESENTATION

We present our case, which is a 37‐year‐old male middle eastern with a past medical history of Hemophilia A. He presented to our outpatient ophthalmology clinic with a right eye sudden decrease in vision for 4 days. On evaluation of the eye, he had features of unilateral panuveitis with para‐macular retinitis, mild vitritis, peripheral punctuate retinal hemorrhage, and macular exudative retinal detachment, findings highly suggestive of Behçet's disease. He was started on a tapering dose of oral prednisolone and referred urgently to the Rheumatology clinic. Upon enquiring, he mentioned a history of recurrent painful oral and genital ulcers for several years for which he did not seek medical attention before. On review of systems, he had no history of joint pain or swelling, no history of skin rash, respiratory or gastrointestinal symptoms, and no history of deep vein thrombosis.

On examination, no oral ulcers or scars were present, but he had one papule on the scrotum. Review of other systems was unremarkable. Blood tests (Table 1) at this point were unremarkable, including autoimmune screening apart from slightly elevated C‐reactive protein (CRP, 17 mg/dl). HLA typing for HLA‐B51 was negative. Pathergy test was negative. A clinical diagnosis of Behçet's disease was made based on history of recurrent painful oral ulcers, genital ulcers, and panuveitis. Hence, immunosuppressive therapy was started with prednisolone, azathioprine, and infliximab.

**TABLE 1:** Laboratory blood tests.

Laboratory test	Result	Normal value
White blood cell count	9.4 × 10^3^/μl	4–10 × 10^3^/μl
Hemoglobin	15.4 g/dl	13–17 g/dl
Platelet	336 × 10^3^/μl	150–400 × 10^3^/μl
Urea	4.8 mmol/L	3.2–7.4 mmol/L
Creatinine	86 μmol/L	64–110 μmol/L
HbA1C %	5.4%	4.8%–5.9%
C‐ reactive protein	17 mg/L	0–5 mg/L
Antinuclear antibody (ANA)	Negative	Negative
Antineutrophil cytoplasmic antibody (ANCA)	Negative	Negative
Anti‐cardiolipin antibody IgM & IgG antibodies	Negative	Negative
Ani‐B2 glycoprotein IgM & IgG antibodies	Negative	Negative
Treponema pallidum antibody	Negative	Negative
QuantiFERON TB plus	Negative	Negative
HLA – B51 typing	Negative	Negative

After a few weeks of treatment, his vision improved with a resolution of signs of uveitis. He was maintained on azathioprine and infliximab, with the latter stopped after 15 months.

**FIGURE 1:**
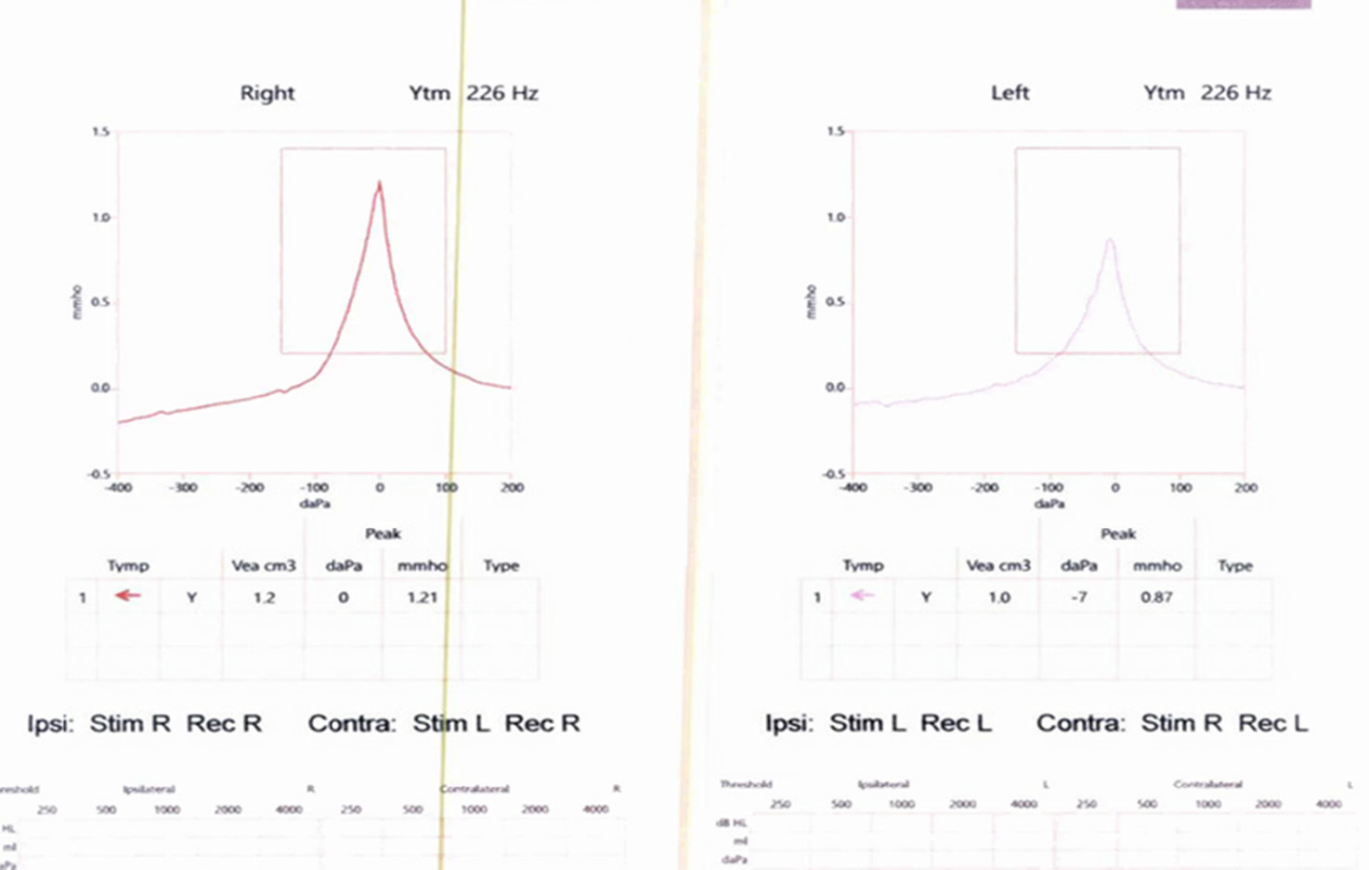
Pure tone audiogram showed bilateral sensorineural hearing loss more on high frequencies.

**FIGURE 2:**
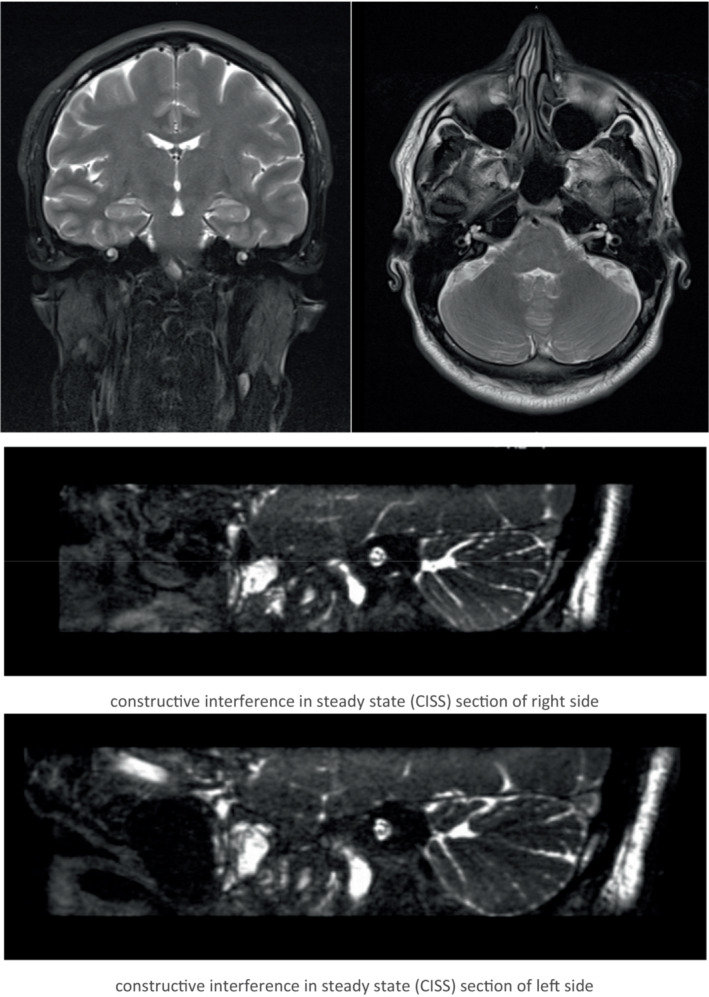
MRI sections of head and internal auditory meatus.

The patient was referred after 7 months to an audiology clinic for evaluation of tinnitus and hearing loss. Symptoms of hearing loss had started in left ear almost at the time of panuveitis but it stabilized after starting immunosuppression and later involved the right ear, few months after stopping Infliximab. Physical examination of the ears was normal, with intact tympanic membrane bilaterally. A pure tone audiogram showed bilateral sensorineural hearing loss more on high frequencies (Figure 1). Magnetic resonance imaging (MRI) of the head and internal auditory meatus was done and was normal (Figure 2). All vestibular tests including video‐nystagmography, oculomotors and post headshake test, computerized dynamic posturography, caloric testing, vestibular evoked myogenic potential and video head impulse test were unremarkable. Based on the previous diagnosis of Behçet's disease, the sensorineural hearing loss was attributed to cochlear involvement by autoimmune inner ear disease related to Behçet's disease. The patient was kept under close follow‐up by Rheumatology, Ophthalmology, and Audiology. He was started on oral steroid prednisolone 70 mg per day for 1 month in addition to 4 doses of intratympanic steroid injections, which partially relieved symptoms. However, he continued to have repeated hearing loss episodes whenever steroid doses were tapered. Infliximab was resumed as a steroid‐sparing agent with a dose of 5 mg/kg every 6 weeks, along with azathioprine 100 mg per day and it helped to successfully taper and stop steroid. The patient was followed for the next 2 years, and his symptoms were controlled with stable audiogram levels.

## 3 DISCUSSION

Behcet's disease is a type of vasculitis that affects arteries and veins of any size. Its presentation commonly involves the mucus membranes, skin, and vasculature. It has an autoimmune etiology after exposure to specific environmental or infectious agents in genetically prone people. The otologic manifestations of Behcet's disease include anything from tinnitus to sensorineural deafness.^3^

Our case was a known Behcet's disease gentleman who presented with recurrent episodes of sensorineural hearing loss, which was attributed to an autoimmune pathology because of his Behcet's disease.

Literature review using Pubmed search has shown multiple case series and case reports of sensorineural deafness as a manifestation in patients with Behcet's disease. A retrospective review of 33 patients with Behcet's disease in tertiary hospital in Spain showed 5 (15%) of total patients had audiovestibular symptoms (including sensorineural hearing loss, vertigo, and bilateral vestibular dysfunction).^3^ Another study which included 63 subjects with Behcet's disease and 63 sex and age‐matched healthy subjects as a control group showed that 35 patients (55%) had hearing loss equal or more than 30 Decibell (DB) and 24 of the 35 patients (68% of the patients with hearing loss) showed hearing loss at higher frequencies of (6–8 kHz).^4^ In another study which recruited 62 Behcet's disease patients and compared with 62 healthy subjects, around 32% (20 of the 62 Behcet's patients) had evidence of sensorineural deafness (at 25 DB in at least two frequencies) by pure tone audiogram, all of the 20 patients with hearing loss had cochlear involvement evidenced by the recruitment investigation tests and auditory brain stem response. 15/20 patients with hearing loss had HLA‐B51 antigen‐positive.^5^

In a prospective placebo‐controlled study that included 29 patients with Behcet's disease and 28 normal subjects, pure tone audiometry (PTA) and transient evoked otoacoustic emission (TEOAE) were determined, and it was found that 10 out of the 29 patients with Behcet's disease had sensorineural hearing loss (34.5%) at 1, 2, 3, 4, and 8 kHz (*p* < 0.498) and this was statistically significant compared to the control group. The difference in TEOAE levels that revealed sound to noise ratio between the two groups was statistically not significant at 1, 1.5, 2, and 3 but was significant at 4 kHz (*p* < 0.02), and the difference was reproducible (*p* < 0.03).^6^

In a review, 24 Behcet's disease patients with 24 age and sex‐matched healthy subjects as control groups were studied to investigate hearing loss in Behcet's disease. It showed that 15 of the 24 subjects with Behcet's disease had sensorineural deafness.^7^

In another study of 35 subjects with Behcet's disease compared with 35 healthy subjects as a control group and were followed up for a year, 28 patients with Behcet's disease (80%) showed some degree of hearing loss.^8^

Some case reports about the association between Behcet's disease and sensorineural hearing loss were also found in the literature.^9^, ^10^, ^11^

A possible pathophysiological explanation for the sensorineural hearing loss in Behcet's disease includes autoimmune inflammation of the common cochlear artery affecting primarily the cochlea's outer hair cells resulting in decreased sensitivity and impaired sound‐evoked neural activity.^8^

Anti‐TNF is usually used in refractory Behcets disease manifestations, mainly sight‐threatening disease with frequent relapse of uveitis in addition to other refractory manifestations,^12^ but its use in sensorineural hearing loss is less well established.

Possible treatment options for the sensorineural loss in Behcet's disease include high‐dose systemic steroids initially followed by steroid‐sparing agents, intratympanic steroid injections, and hyperbaric O_2_ therapy.^9^, ^10^ Initial management for our patient was with oral prednisolone 70 mg and intratympanic steroid injection, which successfully controlled his symptoms with partial improvement and decreased the acute attacks rate. However, the symptoms recurred once the steroid dosage was reduced. So, we started him on infliximab and later azathioprine was added, both successfully helped to control his symptoms and discontinue steroid.

## 4 CONCLUSION

Clinicians should keep in mind that Behcet's disease is not uncommonly associated with sensorineural hearing loss, and that anti‐TNF and steroid‐sparing agents could help control the disease if other modalities fail. Further studies are needed to prove the efficacy of anti‐TNF in this setting.

## AUTHOR CONTRIBUTIONS

Mohanad Faisal involved in manuscript writing, literature review, and application. Haydar Barjas and Wisam Alwassiti involved in manuscript writing and literature review. Walid Omer and Nabeel Abdulla involved in manuscript writing, literature review, and clinical follow‐up.

## CONFLICT OF INTEREST

The authors have no conflict of interest to declare.

## CONSENT

Written informed consent was obtained from the patient for the publication of this case report.

## Data Availability

Data available upon request.
